# A Possible Protective Effect of IgA Against Severe Acute Respiratory Syndrome Corona Virus 2 (SARS-CoV-2) in Bronchoalveolar Lavage in COVID-19 Patients Admitted to Intensive Care Unit

**DOI:** 10.3390/v16121851

**Published:** 2024-11-28

**Authors:** Mariantonietta Di Stefano, Lucia Mirabella, Antonella Cotoia, Giuseppina Faleo, Michela Rauseo, Anna Chiara Rizzo, Josè Ramon Fiore, Gilda Cinnella, Gaetano Serviddio

**Affiliations:** 1 Department of Surgical and Medical Science, Section of Infectious Diseases, University of Foggia, 71122 Foggia, Italy; giuseppina.faleo@unifg.it (G.F.); jose.fiore@unifg.it (J.R.F.); 2 Anesthesia and Intensive Care Unit, Department of Surgical and Medical Science, Policlinico Riuniti di Foggia, University of Foggia, 71122 Foggia, Italy; lucia.mirabella@unifg.it (L.M.); antonella.cotoia@unifg.it (A.C.); michela.rauseo@unifg.it (M.R.); annachiararizzo91@gmail.com (A.C.R.); gilda.cinnella@unifg.it (G.C.); 3 C.U.R.E. (University Center for Liver Disease Research and Treatment), Liver Unit, Department of Medical and Surgical Sciences, University of Foggia, 71122 Foggia, Italy; gaetano.serviddio@unifg.it

**Keywords:** SARS-CoV-2, anti-SARS-CoV-2 IgA, anti-SARS-CoV-2 IgG, bronchoalveolar lavage (BAL), SOFA score, APACHE II score

## Abstract

SARS-CoV-2 infection induces a humoral immune response, producing virus-specific antibodies such as IgM, IgG, and IgA. IgA antibodies are present at mucosal sites, protecting against respiratory and other mucosal infections, including SARS-CoV-2, by neutralizing viruses or impeding attachment to epithelial cells. Since SARS-CoV-2 spreads through the nasopharynx, the specific IgAs of SARS-CoV-2 are produced quickly after infection, effectively contributing to virus neutralization. Dimeric IgA has been reported to be 10 to 15 times more potent than its equivalent IgG, suggesting that this isotype may be particularly interesting in developing new monoclonal antibodies and/or new vaccines efficiently neutralizing the virus at the mucosal sites. It is still unclear whether IgA antibodies in BAL might play a role in the disease course and if their presence may have a prognostic significance. However, a harmful effect on diseases with high IgA titers has been reported. This study evaluated mucosal-specific IgA and IgG profiles in BAL of patients with COVID-19 acute respiratory failure admitted to the ICU. We included 57 patients (41 males and 16 females), admitted to the ICU of the University of Foggia. We used a commercially available ELISA assay to evaluate the presence of SARS-CoV-2 IgG and IgA antibodies in plasma and BAL of the 57 hospitalized patients with severe COVID-19 respiratory failure. However, 40/57 BAL and plasma from infected patients were available for the ELISA test; the remaining specimens were unsuitable. IgG and IgA antibodies against SARS-CoV-2 were detectable in 37 (92.5%) and 40 (100%) plasma specimens, respectively. IgG antibodies were found in a single sample, while IgAs were detected in 19 of 40 BAL samples analyzed. Correlations between these parameters and patient outcomes reveal a signature associated with survival. Interestingly, a statistically significant inverse correlation was found between the mortality rate and the presence of IgA to SARS-CoV-2 in BAL specimens. None of the 19 patients with a positive IgA died, compared to 7 out of 12 patients with a negative IgA-BAL (*p*: <0.0004). Despite being limited in size, this study suggests a significant protective effect of mucosal immunity in COVID-19 patients, even in advanced disease stages, and a role of IgA in the defense against the virus, as well as the possible use of effective vaccines and therapeutic strategies based on IgA antibodies.

## 1. Introduction

SARS-CoV-2 infection induces a humoral immune response, producing virus-specific antibodies such as IgM, IgG, and IgA.

IgA antibodies in mucosal sites protect against respiratory and other mucosal infections by neutralizing viruses or impeding attachment to epithelial cells [1,2]. The immunoglobulin distribution on the mucosa differs from that in serum: In serum, IgG is the dominating isotype (75–80% of serum immunoglobulins) followed by IgA (15%) and IgM (10%) [3]. In contrast, on the mucosa, IgA is the predominant class (~74% of all mucosa immunoglobulin) followed by IgG (~25%) and IgM (~2%) [3]. The overall production of IgA (40–60 mg/kg per day) is higher than all other isotypes [3]. Consistently, with the fact that SARS-CoV-2 spreads through the nasopharynx, the specific IgAs of SARS-CoV-2 are produced quickly at this level after infection, contributing effectively to the neutralization of the virus. Dimeric IgAs have been reported to be 10 to 15 times more potent than their equivalent IgG, suggesting that this isotype may be particularly interesting in developing either new treatment strategies by plasma-derived or monoclonal antibodies and/or new vaccine approaches based on IgA production that can neutralize the virus [3,4].

IgA can be found in the early phase of SARS-CoV-2 infection [5,6]. However, the role of mucosal IgA in response to SARS-CoV-2 infection is still unclear. In mice, vaccination with SARS-CoV-2 proteins induces localized and systemic SARS-CoV-2 challenge suggesting that mucosal-induced IgA is protective [7].

Kim and collaborators generated a recombinant adenovirus-based vaccine candidate against MERS-CoV inducing IgG and secretory IgA and providing long-lasting neutralizing immunity [8]. Recently, two intramuscular vaccinations with mRNA-based vaccines induced systemic circulating IgA anti-FLS and anti-RBD response against SARS-CoV-2 [9].

In this study, higher IgA anti-SARS-CoV-2 S levels were associated with protection from breakthrough infection [9].

From a clinical point of view in a large-scale cohort, it was demonstrated that subjects with selective IgA deficiency have higher rates of COVID-19 disease and re-infection, which demonstrated a very likely role of mucosal immunity in protecting against SARS-CoV-2 [10,11].

On the other hand, data from Ruiz [12] revealed that the antibody persistence, and not the amount, may play an adverse role in COVID-19 pathogenesis; in fact, analyses of IgG and IgA response varied with the patient’s outcome.

S2-specific IgG resulted higher in survivors than non-survivors, whereas S1-specific IgA and sustained levels of S1, RBD, S2, and NP-specific IgG once SARS-CoV-2 was cleared from the lungs were associated with fatal outcomes. The presence of these antibodies in BAL samples in non-survivor patients persists, suggesting that persistent spike- and NP-specific antibodies are non-neutralizing [12].

Although in the early phase of the infection, much of the neutralizing activity in plasma is in samples with IgM and IgA fraction [13,14]. However, IgG antibodies to the spike protein are present at the highest level in circulation after several months compared to IgA and IgM antibodies that decline rapidly after the first-month post-outbreak of the disease.

It is still unclear whether the IgA antibodies at the mucosal site, in the lung, that we can explore by BAL evaluation, might play a role in the course of the disease and if their presence may have a prognostic significance.

The study aimed to evaluate the profile of the mucosal-specific IgA and IgG in BAL of patients with COVID-19 acute respiratory failure admitted in the ICU. Correlations between these parameters and patient clinical outcomes reveal a signature associated with survival.

## 2. Material and Methods

### 2.1. Study Population

Fifty-seven patients were admitted to the ICU of the University of Foggia with a diagnosis of acute respiratory failure, between March 2021 and May 2021. All patients had a severe acute respiratory syndrome coronavirus 2 infection confirmed by the virus in RT PCR from nasopharyngeal swabs, intubation, mechanical ventilation, and a diagnosis of COVID-19. The patients with SARS-CoV-2-negative pneumonia were used as negative controls.

Recorded data included demographics [age, gender, and body mass index (BMI)], comorbidities, previous pharmacological treatments, disease chronology [time from onset of symptoms and hospital admission to initiation of respiratory support, and ICU length of stay (LOS)], symptoms at ICU admission, vital signs [temperature, mean arterial pressure (MAP), and heart rate], laboratory parameters (blood test, coagulation, and biochemical), non-respiratory sequential organ failure assessment (non-respiratory SOFA) and APACHE II scores [15,16], and hospital mortality. The patients were managed according to the recommendations for COVID-19-induced acute respiratory failure.

### 2.2. IgA and IgG from BAL and Serum of Patients

About 57 BAL from SARS-CoV-2-infected and 10 from non-infected individuals were collected in the ICU and processed as described elsewhere [17]. IgA against a recombinant S1 domain of the SARS-CoV-2 was detected using Anti-SARS-CoV-2-ELISA IgA and IgG (Euroimmun Medizinische Laboradiagnostika, Luebeck, Germany, EI 2606-9601A IgA, EI 2606-9601G).

The enzyme-linked immunosorbent assay (ELISA) provides semi-quantitative in vitro determination of human antibodies of immunoglobulin classes IgA and IgG against SARS-CoV-2 (The EUROIMMUN Anti-SARS-CoV-2 Assay), which was used as previously described [18]

Chi-Square and Fisher tests were used to investigate the relationship between the presence of IgA antibodies in SARS-CoV-2-infected patients and age, comorbidities, the SOFA and APACHE II scores, and survival. A *p*-value of less than 0.05 was considered significant.

## 3. Results

The clinical characteristics of the patients enrolled in the study are summarized in Table 1.

Fifty-seven patients were included, 41 males (72%) and 16 females (28%). The enrolled population had a mean age of 70.04 years ± 9.43 years.

Among our study group, all patients had comorbidities such as diabetes, hypertension, obesity, cardiovascular diseases, COPD (chronic obstructive pulmonary disease), allergy, renal failure cancer, asthma, and liver disease.

Of the study group, 22 of 57 infected patients had direct access to the ICU.

Thirty-five patients (61%) were initially referred to the Infectious Diseases, Pneumology, or Internal Medicine department and subsequently transferred to the ICU when clinical conditions worsened.

We assessed the presence of IgG and IgA antibodies recognizing the SARS-CoV-2 S1 protein in both BAL and plasma samples from 40 of 57 infected patients. Anti-SARS-CoV-2 IgG and IgA were detected in 37 (91.5%) and 40 (100%) plasma samples, respectively. In addition, anti-SARS-CoV-2 IgG was detected in one BAL sample (2.5%), while IgA antibodies were present in the BAL samples of 19 patients.

We evaluated the recovery rates for anti-SARS-CoV-2 IgA among a group of patients who were monitored at two time points. In two patients, IgA antibodies were detected in both sequential BAL samples. A patient showed the absence of antibodies in the first sample followed by an increase in IgA antibodies in the second sample. In four patients, however, IgA antibodies were not detected in any BAL samples.

There was no significant correlation (*p* ≥ 0.5) between patients with higher APACHE II scores and the absence of IgA antibodies.

Furthermore, no differences were found between populations with and without IgA and a lower SOFA score. A patient with a higher SOFA score and with anti-SARS-CoV-2-IgA had a fatal outcome.

A significant correlation between the presence of anti-SARS-CoV-2 IgA and the survival of the patients entering the ICU was demonstrated (*p*-value: <0.000496).

The age of the patients played a role in the development of the anti-SARS-CoV-2 IgA; in fact, the data indicated an absence of antibodies in the patients with a higher age (*p*-value < 0.013).

Most patients survived if their APACHE II scores were 9 or less during the first 48 h. Patients with APACHE II scores of 13 or more were at a high risk of death. The sensitivity rate at admission is between 34% and 70%, and the specificity rate is between 76% and 98%.

Mortality is more likely to occur if the SOFA score is higher. Regarding the SOFA score, the maximum score in our population was 10.

**Figure 1:**
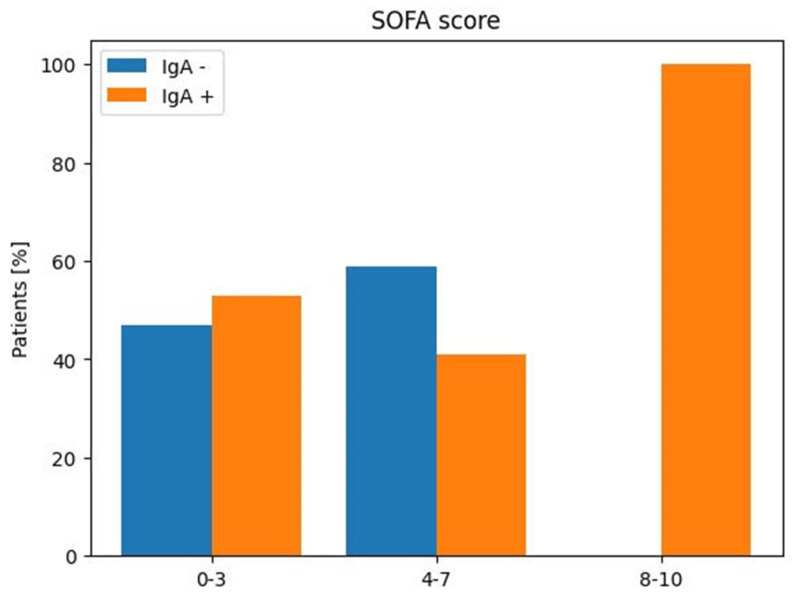
SARS-CoV-2 IgA in BAL according to SOFA score in infected patients enrolled in ICU.

**Figure 2:**
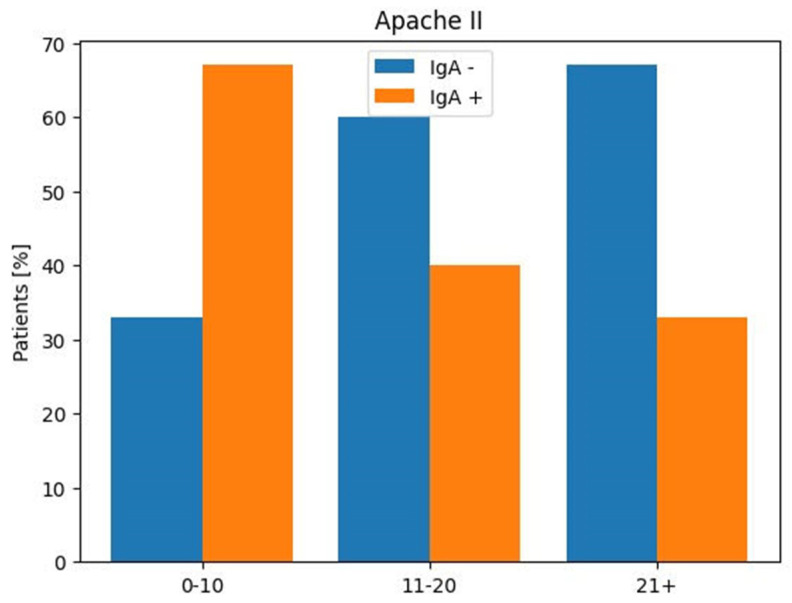
SARS-CoV-2 IgA in BAL according to APACHE II score in infected patients enrolled in ICU.

Regarding APACHE II: 14 (24%) patients had a score between 0 and 10. Of those, 8 showed anti-SARS-CoV-2 IgAs in BAL; 38 (67%) had a score between 11 and 20, and 10 were positive for the presence of IgA antibodies. Only five individuals (9% of the population) had a score higher than 21, and one showed IgA antibodies in BAL (Table 2, Figure 1 and Figure 2).

Regarding the SOFA, 26 patients (46%) showed a score between 0 and 3, and 9 were positive for IgA antibodies. Of 29 patients, 9 with scores between 4 and 7 were positive for antibodies. Only one patient with a score between 8 and 10 displayed the presence of IgA in BAL.

## 4. Discussion

Little information is available on the immune response during SARS-CoV-2 infection at the mucosal level, including the respiratory tract where the virus enters and replicates.

It has been reported that mucosal IgAs play an important role in the defense against both COVID-19 and influenza infections by providing mucosal immunity neutralizing the viruses, preventing viral entry, and reducing their transmission [19,20]. IgA antibodies are produced by plasma cells in the mucosal-associated lymphoid tissues (MALTs) and are secreted into the respiratory tract [21].

In humans, IgAs are present in two subclasses: IgA1 (mainly present in serum) and IgA2 (mainly observed at the mucosal level). Although mucosal (secretory) IgA is dimeric, serum IgA is composed mainly of monomers. However, they also differ in their hinge region and the glycosylation sites. IgA2 antibodies play a role in the pro-inflammatory activation of neutrophils and macrophages concerning the IgA1s. In serum, IgA1 is the principal, while in the mucosa, IgA2s are more present [22,23,24].

We know that individuals affected by selective IgA deficiency (sIgAD) (the most common primary immunodeficiency disorder) were suggested to be at higher risk of severe COVID-19, which is much stronger evidence of disease susceptibility [11]. This seems to emphasize the potential prognostic role of IgA in SARS-CoV-2 infection. According to Padoan and collaborators [25], the IgA response appears and grows early, with a stronger and more persistent response than the IgM response. The authors, further, suggest performing additional longitudinal research on the function and protection efficacy of virus-specific antibodies over time [25].

In the BAL, secretory IgA and IgG are produced locally before entering the alveolar space, and their antigenic repertoire is different from that of the serum humoral response [26,27,28]. Few reports are nevertheless still available about the presence of IgA antibodies in COVID-19 disease [29,30].

In this study, we investigated the presence of anti-SARS-CoV-2 IgG and IgA in both plasma and BAL specimens of hospitalized patients with severe pneumonia who were subjected to oxygen therapy, which was replaced by orotracheal intubation (IOT).

Anti-SARS-CoV-2 IgG was observed in 1 of 40 BAL samples (2.1%), while IgA antibodies were found in 19 of 40 (47.5%) patients’ BAL specimens. These data indicate that IgA to SARS-CoV-2 can be found in the lung mucosa in the later stages of COVID-19, which may contribute to viral clearance.

We observed that the detection of IgA in BAL was significantly correlated with survival, which is contrary to the results from Ruiz and collaborators [12] who reported IgA presence in BAL as a negative prognostic factor. Differences in patient selection, different pandemic periods (2020 vs 2021), different circulating viral variants, possible interference from vaccination initiated, and the use of antivirals could partly explain these discrepancies.

In addition, anti-SARS-CoV-2 IgG and IgA were detected in 37 (91.5%) and 40 (100%) plasma samples, respectively. Although we did not examine the timing of the appearance of IgA and IgG, we are in line with the results by Zervous and collaborators who demonstrated IgG and IgA positivity in most patients tested, with an increase of 100% on day 21 for IgG while IgA antibodies were highest in patients with a severe and critical illness [31].

Furthermore, a study demonstrated a decrease in serum IgA in COVID-19 patients with pneumonia compared to patients with a mild course of disease [2], supporting the important role of these antibodies’ mediated immunity for disease outcome.

SIgA plays an outstanding role in mucosal immunity and its function is impaired in several inflammatory lung diseases [3]. Therefore, supplementation with therapeutic IgA antibodies [32,33] might be a worthwhile strategy. In conclusion, our findings, although limited in size, support the protective role of IgA immune response in COVID-19 patients in survival, even in advanced stages of the disease. Furthermore, we suggest continuing the study to designate IgA as a promising therapeutic agent for the treatment of inflammatory lung diseases.

## Figures and Tables

**Table 1:** Clinical demographic data.

57 Patients		Anti SARS-CoV-2-IgA	Survivors	Not Survived
		Positive in BAL		
Mean age	70.04 ± 9.43 years			
Caucasian	All			
Males	41	31	15	16
Females	16	9	4	5
Comorbidities				
Diabetes			7	14
Arterial hypertension			16	15
Obesity			9	7
Cardiovascular diseases			7	6
COPD			2	3
Allergy			2	3
Renal failure/cancer			1	1
Asthma				1
Liver diseases				1
Bacterial infection			1	

**Table 2:** The APACHE and SOFA scores in SARS-CoV-2 selected patients tested for the presence of anti-SARS-CoV-2 IgA in BAL.

APACHE II		Anti-SARS-CoV-2 IgA	Anti-SARS-CoV-2 IgA
Score		Positive	Negative
	Patients		
	No (%)	No (%)	No (%)
0–10	14 (24%)	8 (67%)	4 (33%)
11–20	38 (67%)	10 (40%)	15 (60%)
≥21	5 (9%)	1 (33%)	2 (67%)
SOFA		Anti-SARS-CoV-2 IgA	Anti-SARS-CoV-2 IgA
Score		Positive	Negative
	Patients		
	No (%)	No (%)	No (%)
0–3	26 (46%)	9 (53%)	8 (47%)
4–7	29 (51%)	9 (41%)	13 (59%)
8–10	2 (3%)	1 (100%)	0 (0%)

## Data Availability

Data are available on request to the corresponding author.

## References

[1] Montague B.T., Wipperman M.F., Chio E., Crow R., Hooper A.T., O’Brien M.P., Simões E.A.F. (2022). Elevated serum IgA following vaccination against SARS-CoV-2 in a cohort of high-risk first responders. Sci. Rep..

[2] Gupta S.L., Tyagi R., Dhar A., Oswal N., Khandelwal A., Jaiswal R.K. (2023). Children’s SARS-CoV-2 Infection and Their Vaccination. Vaccines.

[3] Bohländer F. (2023). A new hope? Possibilities of therapeutic IgA antibodies in the treatment of inflammatory lung diseases. Front. Immunol..

[4] Pantaleo G., Correia B., Fenwick C., Joo V.S., Perez L. (2022). Antibodies to combat viral infections: Development strategies and progress. Nat. Rev. Drug Discov..

[5] Sterlin D., Mathian A., Miyara M., Mohr A., Anna F., Claër L., Quentric P., Fadlallah J., Devilliers H., Ghillani P. (2021). IgA dominates the early neutralizing antibody response to SARS-CoV-2. Sci. Transl. Med..

[6] Esmat K., Jamil B., Kheder R.K., Kombe A.J., Zeng W., Ma H., Jin T. (2024). Immunoglobulin A response to SARS-CoV-2 infection and immunity. Heliyon.

[7] Izikson R., Brune D., Bolduc J.S., Bourron P., Fournier M., Moore T.M., Pandey A., Perez L., Sater N., Shrestha A. (2022). Safety and immunogenicity of a high-dose quadrivalent influenza vaccine administered concomitantly with a third dose of the mRNA-1273 SARS-CoV-2 vaccine in adults aged ≥65 years: A phase 2, randomized, open-label study. Lancet Respir. Med..

[8] Kim M.H., Kim H.J., Chang J. (2019). Superior immune responses induced by intranasal immunization with recombinant adenovirus-based vaccine expressing full-length Spike protein of Middle East respiratory syndrome coronavirus. PLoS ONE..

[9] Norton N.J., Ings D.P., Fifield K.E., Barnes D.A., Barnable K.A., Harnum D.O.A., Holder K.A., Russell R.S., Grant M.D. (2023). Characteristics of Vaccine- and Infection-Induced Systemic IgA Anti-SARS-CoV-2 Spike Responses. Vaccines.

[10] Magen E., Merzon E., Green I., Golan-Cohen A., Vinker S., Israel A. (2023). Selective IgA deficiency and COVID-19. J. Allergy Clin. Immunol. Pract..

[11] Çölkesen F., Kandemir B., Arslan Ş., Çölkesen F., Yıldız E., Korkmaz C., Vatansev H., Evcen R., Aykan F.S., Kılınç M. (2022). Relationship between Selective IgA Deficiency and COVID-19 Prognosis. Jpn. J. Infect. Dis..

[12] Ruiz M.J., Siracusano G., Cottignies-Calamarte A., Tudor D., Real F., Zhu A., Pastori C., Capron C., Rosenberg A.R., Temperton N. (2022). Persistent but dysfunctional mucosal SARS-CoV-2-specific IgA and low lung IL-1β associate with COVID-19 fatal outcome: A cross-sectional analysis. Front. Immunol..

[13] Gasser R.M., Cloutier M., Prévost J., Fink C., Ducas É., Ding S., Dussault N., Landry P., Tremblay T., Laforce-Lavoie A. (2021). Major role of IgM in the neutralizing activity of convalescent plasma against SARS-CoV-2. Cell Rep..

[14] Verkerke H., Saeedi B.J., Boyer D., Allen J.W., Owens J., Shin S., Horwath M., Patel K., Paul A., Wu S.C. (2021). Are We Forgetting About IgA? A Re-examination of Coronavirus Disease 2019 Convalescent Plasma. Transfusion.

[15] Vincent J.L., Moreno R., Takala J., Willatts S., De Mendonça A., Bruining H., Reinhart C.K., Suter P.M. (1996). Thijs LG. The SOFA (Sepsis-related Organ Failure Assessment) score to describe organ dysfunction/failure. On behalf of the Working Group on Sepsis-Related Problems of the European Society of Intensive Care Medicine. Intensive Care Med..

[16] Knaus W.A., Draper E.A., Wagner D.P., Zimmerman J.E. (1985). APACHE II: A severity of disease classification system. Crit. Care Med..

[17] Delclaux C., Roupie E., Blot F., Brochard L., Lemaire F., Brun-Buisson C. (1997). Lower respiratory tract colonization and infection during severe acute respiratory distress syndrome: Incidence and diagnosis. Am. J. Respir. Crit. Care Med..

[18] Gededzha M.P., Mampeule N., Jugwanth S., Zwane N., David A., Burgers W.A., Blackburn J.M., Grove J.S., George J.A., Sanne I. (2021). Performance of the EUROIMMUN Anti-SARS-CoV-2 ELISA Assay for detection of IgA and IgG antibodies in South Africa. PLoS ONE.

[19] Tyagi R., Basu S., Dhar A., Gupta S., Gupta S.L., Jaiswal R.K. (2023). Role of Immunoglobulin A in COVID-19 and Influenza Infections. Vaccines.

[20] Wang Z., Lorenzi J.C.C., Muecksch F., Finkin S., Viant C., Gaebler C., Cipolla M., Hoffmann H.H., Oliveira T.Y., Oren D.A. (2021). Enhanced SARS-CoV-2 neutralization by dimeric IgA. Sci Transl Med..

[21] Bemark M., Angeletti D. (2021). Know your enemy or find your friend?—Induction of IgA at mucosal surfaces. Immunol. Rev..

[22] de Sousa-Pereira P., Woof J.M. (2019). IgA: Structure, Function, and Developability. Antibodies.

[23] Bakema J.E., van Egmond M. (2011). Immunoglobulin A: A next generation of therapeutic antibodies?. MAbs.

[24] Woof J.M., Russell M.W. (2011). Structure and function relationships in IgA. Mucosal Immunol..

[25] Padoan A., Sciacovelli L., Basso D., Negrini D., Zuin S., Cosma C., Faggian D., Matricardi P., Plebani M. (2020). IgA-Ab response to spike glycoprotein of SARS-CoV-2 in patients with COVID-19: A longitudinal study. Clin. Chim. Acta..

[26] Brandtzaeg P. (2013). Secretory IgA: Designed for Anti-Microbial Defense. Immunol. Front..

[27] Bomsel M., Tudor D., Drillet A.S., Alfsen A., Ganor Y., Roger M.G., Mouz N., Amacker M., Chalifour A., Diomede L. (2011). Immunization with HIV-1 gp41 subunit virosomes induces mucosal antibodies protecting nonhuman primates against vaginal SHIV challenges. Immunity.

[28] Khamassi M., Xu L., Rey J., Duchemin M., Bouceba T., Tuffery P., Tudor D., Bomsel M. (2020). The CH1α domain of mucosal gp41 IgA contributes to antibody specificity and antiviral functions in HIV-1 highly exposed Sero-Negative individuals. PLoS Pathog..

[29] Butler S.E., Crowley A.R., Natarajan H., Xu S., Weiner J.A., Bobak C.A., Mattox D.E., Lee J., Wieland-Alter W., Connor R.I. (2021). Distinct Features and Functions of Systemic and Mucosal Humoral Immunity Among SARS-CoV-2 Convalescent Individuals. Front. Immunol..

[30] Yu H.Q., Sun B.Q., Fang Z.F., Zhao J.C., Liu X.Y., Li Y.M., Sun X.Z., Liang H.F., Zhong B., Huang Z.F. (2020). Distinct features of SARS-CoV-2-specific IgA response in COVID-19 patients. Eur. Respir. J..

[31] Zervou F.N., Louie P., Stachel A., Zacharioudakis I.M., Ortiz-Mendez Y., Thomas K., Aguero-Rosenfeld M.E. (2021). SARS-CoV-2 antibodies: IgA correlates with severity of disease in early COVID-19 infection. J. Med. Virol..

[32] Ejemel M., Li Q., Hou S., Schiller Z.A., Tree J.A., Wallace A., Amcheslavsky A., Kurt Y., Buttigieg K.R., Elmore M.J. (2020). A cross-reactive human IgA monoclonal antibody blocks SARS-CoV-2 spike-ACE2 interaction. Nat Commun..

[33] Göritzer K., Groppelli E., Grünwald-Gruber C., Figl R., Ni F., Hu H., Li Y., Liu Y., Hu Q., Puligedda R.D. (2024). Recombinant neutralizing secretory IgA antibodies for preventing mucosal acquisition and transmission of SARS-CoV-2. Mol. Ther..

